# Suitability of the Current Health Technology Assessment of Innovative Artificial Intelligence-Based Medical Devices: Scoping Literature Review

**DOI:** 10.2196/51514

**Published:** 2024-05-13

**Authors:** Line Farah, Isabelle Borget, Nicolas Martelli, Alexandre Vallee

**Affiliations:** 1 Innovation Center for Medical Devices Department Foch Hospital Suresnes France; 2 Groupe de Recherche et d'accueil en Droit et Economie de la Santé Department University Paris-Saclay Orsay France; 3 Department of Biostatistics and Epidemiology Gustave Roussy University Paris-Saclay Villejuif France; 4 Oncostat U1018, Inserm Équipe Labellisée Ligue Contre le Cancer University Paris-Saclay Villejuif France; 5 Pharmacy Department Georges Pompidou European Hospital Paris France; 6 Department of Epidemiology and Public Health Foch Hospital Suresnes France

**Keywords:** artificial intelligence, machine learning, health technology assessment, medical devices, evaluation

## Abstract

**Background:**

Artificial intelligence (AI)–based medical devices have garnered attention due to their ability to revolutionize medicine. Their health technology assessment framework is lacking.

**Objective:**

This study aims to analyze the suitability of each health technology assessment (HTA) domain for the assessment of AI-based medical devices.

**Methods:**

We conducted a scoping literature review following the PRISMA (Preferred Reporting Items for Systematic Reviews and Meta-Analyses) methodology. We searched databases (PubMed, Embase, and Cochrane Library), gray literature, and HTA agency websites.

**Results:**

A total of 10.1% (78/775) of the references were included. Data quality and integration are vital aspects to consider when describing and assessing the technical characteristics of AI-based medical devices during an HTA process. When it comes to implementing specialized HTA for AI-based medical devices, several practical challenges and potential barriers could be highlighted and should be taken into account (AI technological evolution timeline, data requirements, complexity and transparency, clinical validation and safety requirements, regulatory and ethical considerations, and economic evaluation).

**Conclusions:**

The adaptation of the HTA process through a methodological framework for AI-based medical devices enhances the comparability of results across different evaluations and jurisdictions. By defining the necessary expertise, the framework supports the development of a skilled workforce capable of conducting robust and reliable HTAs of AI-based medical devices. A comprehensive adapted HTA framework for AI-based medical devices can provide valuable insights into the effectiveness, cost-effectiveness, and societal impact of AI-based medical devices, guiding their responsible implementation and maximizing their benefits for patients and health care systems.

## Introduction

### Background

Artificial intelligence (AI) has emerged as a transformative technology with vast potential across various sectors, including health care [1,2]. In this field, AI-based medical devices have garnered significant attention due to their ability to revolutionize diagnosis, treatment, and patient monitoring [3]. These devices use advanced algorithms and machine learning techniques to analyze complex medical data sets, thereby providing valuable insights and support to health care professionals in their decision-making. To meet the ever-increasing demand for integration of AI-based medical devices into clinical practice, their efficient evaluation through adapted health technology assessment (HTA) is now crucial [4]. Several frameworks have been published showing an adaptation of items for reporting clinical trials related to AI-based medical devices (Table 1). However, there is still a lack of a much needed adaptation of the standard HTA framework to better suit the assessment of AI-based health care technologies.

**Table 1:** Summary of different frameworks on artificial intelligence (AI)–based health technologies.

Framework	Function	Definition and aim	Domains and items assessed	Reference
HTA^a^ Core Model	Medical device assessment	A systematic evaluation of a medical technology’s clinical, economic, ethical, and social implications to determine its overall value and impact on health care delivery; a methodological framework for production and sharing of HTA information	It evaluates nine domains: (1) health problem and current use of technology, (2) description and technical characteristics of the technology, (3) safety, (4) clinical effectiveness, (5) costs and economic evaluation, (6) ethical analysis, (7) organizational aspects, (8) patients and social aspects, and (9) legal aspects	European Network for Health Technology Assessment [5]
CONSORT-AI^b^	Trial reporting	A set of recommendations for clinical trial reports evaluating interventions with an AI component	It evaluates the 25 CONSORT^c^ 2010 items+14 AI-specific extension items	Liu et al [6]
SPIRIT-AI^d^	Trial reporting	New reporting guidelines for clinical trial protocols evaluating interventions with an AI component	It evaluates the 33 SPIRIT^e^ 2013 items+ 15 AI-specific extension items	Rivera et al [7]
TRIPOD-AI^f^	Research reporting	Reports of research or endeavors in which a multivariable prediction model is being developed (or updated) or validated (tested) using any (supervised) machine learning technique	It evaluates the 22 TRIPOD^g^ 2013 items and the AI extension item definitions	Collins et al [8]

^a^HTA: health technology assessment.

^b^CONSORT-AI: Consolidated Standards of Reporting Trials–Artificial Intelligence.

^c^CONSORT: Consolidated Standards of Reporting Trials.

^d^SPIRIT-AI: Standard Protocol Items: Recommendations for Interventional Trials–Artificial Intelligence.

^e^SPIRIT: Standard Protocol Items: Recommendations for Interventional Trials.

^f^TRIPOD-AI: Transparent Reporting of a Multivariable Prediction Model for Individual Prognosis or Diagnosis-Artificial Intelligence.

^g^TRIPOD: Transparent Reporting of a Multivariable Prediction Model for Individual Prognosis or Diagnosis.

An HTA involves a systematic evaluation of a medical technology’s clinical, economic, ethical, and social implications to determine its overall value and impact on health care delivery [9]. The European Network for Health Technology Assessment has designed an HTA Core Model that provides a methodological framework for production and sharing of HTA information [5]. It evaluates the following nine domains: (1) health problem and current use of technology, (2) description and technical characteristics of the technology, (3) safety, (4) clinical effectiveness, (5) costs and economic evaluation, (6) ethical analysis, (7) organizational aspects, (8) patients and social aspects, and (9) legal aspects (Multimedia Appendix 1).

A full understanding of the capabilities, limitations, and specificities of AI-based medical devices is paramount to complete these HTA domain assessments and, thereby, inform evidence-based decision-making and allow for policy development and the responsible integration of these technologies into health care systems [10,11]. Much uncertainty remains with regard to the reliability of AI-based medical devices, data issues, and regulatory processes, resulting in multiple challenges faced by HTA agencies assessing new technologies and delivering their approval [4]. Nevertheless, AI-based medical devices require strict regulations and specific legislations [12-14]. Over the last few decades, the evaluation of AI-based medical devices through an HTA process has received growing interest, as shown by the increase from 1 published article in 1990 to 94 in December 2023, with 484 articles in total during the period 1990-2023 (Table 2).

**Table 2:** Occurrences of “technology assessment” AND “medical device” AND “artificial intelligence” from inception to December 2023 in the PubMed literature.

Year	Articles (n=484), n (%)
1990-1999	5 (1%)
2000-2009	44 (9%)
2010-2019	151 (32%)
2020-2023	273 (58%)

### Objectives

The objective of this review was to critically assess the comprehensive suitability of the current HTA process for AI-based medical devices. By evaluating the performance and capabilities of AI-based medical devices across multiple dimensions, this review aimed to inform health care professionals, policy makers, and researchers about the challenges and opportunities associated with these technologies. Ultimately, this review sought to facilitate evidence-based decision-making; promote responsible implementation; and maximize the potential benefits of AI-based medical devices in improving health care quality, accessibility, and outcomes.

To this end, we analyzed the suitability of each HTA domain for the assessment of AI-based medical devices and proposed an adapted HTA framework.

## Methods

### Search Strategies

A scoping literature review was conducted following the PRISMA (Preferred Reporting Items for Systematic Reviews and Meta-Analyses) methodology [15,16]. All articles related to HTA methods for AI-based medical devices were selected. Data extraction focused on assessment criteria, methodological evaluations, and results.

The search terms and strategy outlined in Textbox 1 were used for the scoping review.

**Search strategies and Medical Subject Heading (MeSH) terms used for the scoping literature review.**

**Database and search strategy**

- PubMed
  Health technology assessment
    “technology assessment, biomedical” [MeSH term] OR (“technology” [all fields] AND “assessment” [all fields] AND “biomedical” [all fields]) OR “biomedical technology assessment” [all fields] OR (“health” [all fields] AND “technology” [all fields] AND “assessment” [all fields]) OR “health technology assessment” [all fields]
  Medical device
    “equipment and supplies” [MeSH term] OR (“equipment” [all fields] AND “supplies” [all fields]) OR “equipment and supplies” [all fields] OR (“medical” [all fields] AND “device” [all fields]) OR “medical device” [all fields]
  Artificial intelligence
    “artificial intelligence” [MeSH term] OR (“artificial” [all fields] AND “intelligence” [all fields]) OR “artificial intelligence” [all fields]
- Embase
  (“artificial intelligence” OR “artificial intelligence-based medical device”) AND “technology assessment”
- Cochrane Library
  (technology assessment) and (artificial intelligence)

### Databases

We searched multiple databases: PubMed, Embase, and the Cochrane Library. Additional articles were retrieved manually from the gray literature and from HTA agency websites (Bundesinstitut für Arzneimittel und Medizinprodukte [Federal Institute for Drugs and Medical Devices in Germany], Haute Autorité de Santé [French Health Authority], National Health Service, National Institute for Health and Care Excellence, National Institute of Public Health, International Network of Agencies for Health Technology Assessment, and the European Network for Health Technology Assessment).

### Study Selection Process

The studies were selected by 2 reviewers (LF and AV). After removing duplicates, both reviewers independently screened the abstracts to select eligible articles and then analyzed full-text reports for eligibility. A third party (NM) resolved the possible discrepancies highlighted during the selection process should a consensus not be reached. An extraction database was used to list the selected articles meeting the inclusion criteria to ensure that all eligible articles were included.

The inclusion and exclusion criteria were related to (1) language (this review was limited to the English and French languages), (2) article type (reviews and primary research were included, whereas other article types were excluded, such as abstracts, commentaries, editorials, letters to the editor, case reports, case series, animal studies, phase-1 and phase-2 studies, pilot studies, duplicate studies, irrelevant studies, studies with the wrong aim, and studies available in abstract form only), (3) type of technology (only AI-based medical devices were eligible), and (4) type of evaluation (HTA articles were included).

### Data Extraction

In total, 2 analysts (LF and AV) independently extracted data items from the selected articles. A third party was involved to resolve any discrepancies highlighted during the selection process. The following data were extracted from each article: (1) general characteristics of the studies (authors, journal, and publication date), (2) study objective, and (3) HTA assessment domain for AI-based medical devices related to the article.

### Methodological Quality Appraisal

Neither the methodological quality nor the risk of bias of the included articles were assessed, consistent with scoping review guidance [15].

### Application Example of the HTA of an AI-Based Medical Device

Concerning each HTA domain, we added a case study of the HTA of several AI-based medical devices in diabetes to illustrate our recommendations.

## Results

### Scoping Review Results

**Figure 1:**
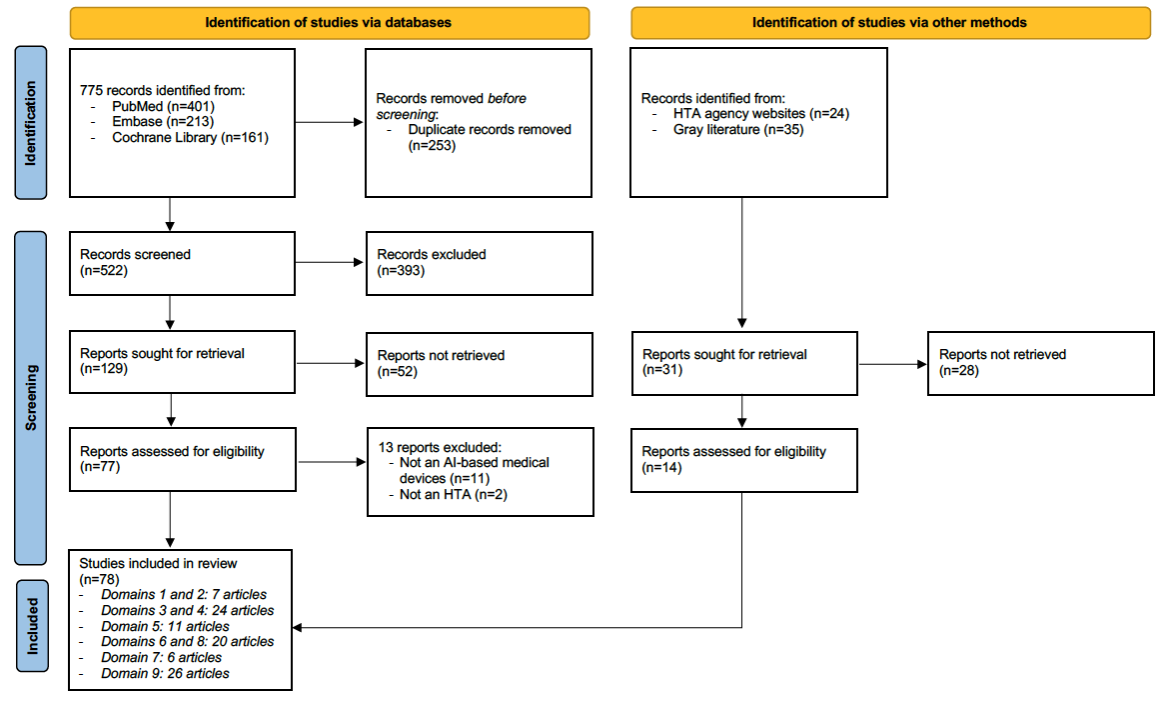
PRISMA (Preferred Reporting Items for Systematic Reviews and Meta-Analyses) flowchart of the study selection process. AI: artificial intelligence; HTA: health technology assessment; MD: medical device.

The literature search resulted in 775 citations summarized in the PRISMA flowchart (Figure 1). After removal of 32.6% (253/775) of duplicates, we carried out an initial screening of the remaining 522 publications, resulting in 129 (24.7%) potentially relevant full-text papers. After further screening, we assessed 77 reports for eligibility, based on which we excluded 11 (14%) for not referring to AI-based medical devices and 2 (3%) for not being HTAs. We then included a further 14 records from 59 potential citations available from HTA agency websites and gray literature.

This gave a total of 78 included articles covering one or multiple HTA domains, the distribution of which is provided at the bottom of Figure 1.

We then summarized the data collection for each selected article in Multimedia Appendix 2 [1,2,4,9,10,17-89].

### Suitability of Each HTA Domain for the Assessment of AI-Based Medical Devices

As domain 1, health problem and current use of technology, is suitable for any type of medical technology and systematically addressed these technologies, we focused on the suitability of the HTA domains (from 1 to 9).

#### Domains 1 and 2 (Health Problem, Current Use, Description, and Technical Characteristics of the Technology)

Data quality and integration are vital aspects to consider when describing and assessing the technical characteristics of AI-based medical devices during an HTA process [17]. These devices often rely on accessing and analyzing diverse health care data sources, including electronic health records, medical images, genetic data, and wearable device data. Therefore, it is essential to evaluate their ability to seamlessly integrate and exchange data with existing health care systems [18].

First, interoperability refers to the ability of AI-based medical devices to interact and communicate with other health care technologies and systems [10]. This includes the ability to access and use data from different sources, such as laboratory systems, imaging archives, and patient health records. The assessment should consider whether the devices adhere to relevant data standards and protocols, ensuring efficient data exchange and compatibility with existing health care infrastructures [19].

Second, data integration involves the ability of AI-based medical devices to aggregate and analyze data from multiple sources to provide comprehensive and accurate insights [20]. The HTA should assess whether the devices can handle different data types, formats, and resolutions and whether they can effectively integrate and harmonize data from disparate sources.

In addition, data privacy and security considerations are crucial when evaluating AI-based medical devices [21]. The assessment should examine whether the devices comply with relevant data protection regulations, use appropriate data anonymization and encryption techniques, and have robust security measures in place to protect patient information [22].

Therefore, a comprehensive HTA should address both the interoperability and data integration capabilities of AI-based medical devices, ensuring that they can seamlessly interact with existing health care systems and integrate data from multiple sources and that they adhere to data privacy and security standards. By evaluating all these aspects, the HTA could determine the devices’ feasibility, scalability, and potential impact on health care delivery.

#### Domain 3 (Safety) and Domain 4 (Clinical Effectiveness)

The assessment of clinical effectiveness and the impact on patient outcomes is a crucial aspect when evaluating AI-based medical devices through a comprehensive HTA [4]. While accuracy and performance metrics are important, it is essential to determine how these devices translate into tangible benefits for patients and health care delivery [23].

Clinical utility refers to the extent to which the AI-based medical device improves clinical decision-making, patient outcomes, and health care processes [24]. The HTA should examine whether the device provides actionable and reliable information that potentially helps health care professionals make more accurate diagnoses or improve treatment plans or monitoring strategies [25,26]. It should also evaluate the potential impact of the device on patient outcomes by assessing, for example, improved survival rates, reduced complications, or enhanced quality of life [27].

To assess clinical utility, the HTA should consider the device’s performance in relevant clinical scenarios and its ability to address specific clinical questions or challenges. This may involve evaluating the device’s performance against established clinical guidelines or expert opinions as well as considering the device’s potential to fill gaps in clinical practice or enhance existing diagnostic or treatment methods.

Furthermore, the HTA should examine the broader impact of AI-based medical devices on health care systems and resource allocation [21]. This includes evaluating the devices’ potential to optimize resource use, reduce health care costs, or improve workflow efficiency. Economic evaluations such as cost-effectiveness analyses can provide insights into the value for money and long-term cost savings associated with the adoption of these devices [28,29].

A comprehensive HTA should thoroughly assess the clinical effectiveness and impact on patient outcomes of AI-based medical devices, examining their performance in relevant clinical scenarios, their alignment with clinical guidelines, and their potential to improve health care processes and resource allocation. By evaluating these aspects, the HTA can provide a holistic understanding of the AI devices’ effectiveness and their potential to positively transform health care delivery [21].

AI-based medical devices must undergo rigorous clinical validation to assess their performance and reliability in real-world health care settings [30,31]. Clinical validation involves evaluating the device’s accuracy, sensitivity, specificity, and overall diagnostic or prognostic performance. In addition, the devices should be tested across diverse patient populations and compared against gold-standard reference methods or expert opinions [32].

Furthermore, the use of real-world evidence (RWE) is crucial for a comprehensive HTA [33]. RWE involves gathering data from routine clinical practice, electronic health records, and other sources to evaluate the device’s effectiveness and safety in real-world settings [34]. These data can help assess the device’s performance in a broader patient population and identify any potential limitations or biases that may arise in specific clinical scenarios.

Robust clinical validation studies and the integration of RWE provide critical evidence for the evaluation of AI-based medical devices in an HTA process [4]. These studies should include a sufficient sample size, appropriate study design, and statistical analysis to ensure the validity and generalizability of the results [35-38]. By considering clinical validation and RWE adapted to AI-based medical devices, HTA can provide valuable insights into their clinical utility and impact in real-world health care settings, facilitating evidence-based decision-making for their adoption and use.

A lack of safety evaluation was highlighted in a systematic review, showing that only 9% of AI-based medical device studies evaluated safety criteria [4]. However, safety is crucial for the confidence in and adoption of AI-based technologies for both patients and health care professionals [39]. While these devices have the potential to improve diagnosis and treatment outcomes, their integration into clinical practice must be accompanied by robust safety evaluations [2,40]. Identifying and mitigating potential risks, such as algorithmic bias, data privacy breaches, and algorithm failures, is essential to protect patient well-being and maintain trust in these innovative technologies [41].

To ensure the safety of AI-based medical devices, it is crucial to establish standardized evaluation frameworks and guidelines [39]. These frameworks should encompass rigorous testing methodologies, validation procedures, and continuous monitoring of AI performance. Collaboration among stakeholders, including manufacturers, regulatory agencies, health care providers, and researchers, is essential to develop and implement comprehensive safety evaluation protocols. Addressing the safety gap in AI-based medical devices not only ensures patient welfare but also instills confidence in health care professionals to embrace and use these technologies effectively [40]. By prioritizing safety evaluation, we can unleash the full potential of AI-based medical devices, leading to transformative advancements in health care delivery [1].

#### Domain 5 (Costs and Economic Evaluation)

Costs and economic evaluation play a crucial role in the comprehensive assessment of health technology adoption, particularly in the context of the HTA of AI-based medical devices [4,9,42]. While AI technologies show huge potential to improve health care delivery, reduce time to diagnosis, and save money, their economic impact requires careful consideration to ensure their successful integration and sustainability [23].

When assessing AI-based medical devices through the HTA process, economic evaluations involve analyzing the costs and benefits associated with the adoption and use of the technology [4]. These evaluations go beyond the upfront costs of acquiring the AI-based medical device; they encompass various factors, such as training, infrastructure modifications, maintenance, and ongoing operational costs [43]. Cost analysis also includes improved patient outcomes, reduced hospital readmissions, and shortened hospital stays. By conducting economic evaluations, decision makers can gauge the cost-effectiveness and cost utility to weigh the affordability of AI-based medical devices for their particular use against the potential mid- and long-term cost savings considered [44].

HTA evaluates the suitability of AI-based medical devices by considering their potential economic benefits, risks, and ethical implications [45,46]. By incorporating economic evaluation into the HTA process, decision makers can make evidence-based choices about the allocation of limited health care resources and prioritize interventions that provide the greatest value for money [4,47].

Finally, considering cost savings and conducting economic evaluations as part of the HTA process helps facilitate the adoption of AI-based medical devices [37]. It provides decision makers with the necessary information to determine the financial feasibility and potential return on investment associated with implementing these technologies. HTA ensures that AI-based medical devices are suitable for integration into the health care system, fostering confidence in their effectiveness, efficiency, and cost utility [4,43,48].

#### Domain 6 (Ethical Analysis) and Domain 8 (Patients and Social Aspects)

Ethical and societal implications are critical aspects that must be considered when evaluating the suitability of AI-based medical devices for a comprehensive HTA [45]. The integration of AI technologies into health care raises important ethical concerns that need to be addressed to ensure responsible and equitable use [49].

Data privacy and patient consent are primary ethical considerations [50]. The HTA should evaluate whether AI-based medical devices adhere to strict data protection regulations, maintain patient confidentiality, and obtain appropriate informed consent for data use. It is essential to assess whether the devices have mechanisms in place to handle sensitive patient information securely and protect it against unauthorized access or data breaches.

Algorithmic fairness and bias are additional ethical concerns [51]. AI algorithms can inadvertently perpetuate biases present in the data used for training, resulting in unequal treatment or access to health care resources [52]. The HTA should assess whether the devices have been evaluated for fairness and bias and consider the steps taken to mitigate any identified biases [53].

Moreover, the HTA should examine the impact of AI-based medical devices on health care disparities and access to care [54]. It is crucial to assess whether the devices have the potential to exacerbate existing inequalities or whether they can contribute to reducing disparities by improving health care access, particularly for underserved populations.

Accountability and transparency in AI decision-making processes are also important ethical considerations [55]. The HTA should evaluate whether the AI devices provide clear explanations for their outputs and ensure that health care professionals and patients can understand and challenge the device’s recommendations [21].

A comprehensive HTA should thoroughly examine the ethical and societal implications of AI-based medical devices, ensuring that they prioritize patient privacy, fairness, and equitable access to care; fostering trust; and ensuring that these devices align with societal values and goals.

To ensure the adoption of AI-based medical devices through a truthfully AI concept, explainability, interpretability, and transparency are important considerations for their comprehensive HTA [56]. Indeed, these devices often use complex algorithms and machine and deep learning techniques, resulting in their operating as “black boxes” that reach decisions and recommendations that are challenging to understand [55]. However, explainability and transparency are essential to ensure trust, accountability, and acceptance of AI-based technologies in health care. First, Explainability refers to the ability to understand and interpret the reasoning behind the device’s outputs [56]. It involves providing clinicians and users with transparent explanations of how the AI algorithm processes input data and generates results. This enables health care professionals to trust the device’s recommendations and make informed decisions based on the provided information. The HTA should evaluate the extent to which AI-based medical devices can provide interpretable and understandable explanations of their decision-making processes [17,46]. Second, Transparency involves disclosing important information about the AI-based medical devices, including the data used for training, algorithmic methodologies used, and potential limitations or biases [57]. Transparency promotes trust and allows stakeholders to assess the device’s reliability and potential risks. The HTA should assess whether the device manufacturers provide clear documentation and information to health care professionals and patients about the device’s capabilities, limitations, and potential errors [21,58,59]. Transparency is closely linked to regulatory considerations [60,61]. The HTA should consider whether the device complies with relevant regulatory standards and whether the manufacturers have provided the necessary documentation and evidence to support their ethical claims [21,62,63].

A comprehensive HTA process should address concerns regarding the explainability and transparency of AI-based medical devices, trust, accountability, and ethical implications related to the use of AI in health care [45,46].

#### Domain 7 (Organizational Aspects)

Organizational aspects play a crucial role in the effective integration of AI-based medical devices into health care systems [64]. To ensure consistency, transparency, and comparability in the evaluation process, there is a growing need for a robust methodological framework that provides standardized guidelines for assessing the organizational impact of the implementation of AI-based medical devices [65]. According to a descriptive analysis led in German hospitals, the main barriers to AI-based medical device adoption were lack of resources (staff, knowledge, and financial) [66].

Clear indicators are needed to measure the organizational readiness for and impact of AI-based medical devices [4,10,67]. Several criteria have been highlighted, such as (1) health care workplace readiness and stakeholder acceptance, (2) AI-based medical device organization alignment assessment, and (3) business plan (financing and investments) [64].

#### Domain 9 (Legal Aspects)

The roles and responsibilities of health care professionals are also impacted by these AI solutions [68]. It is necessary to evaluate whether they complement or replace health care professionals’ expertise and whether additional training, supervision, or support is required for their optimal use [69,70]. As AI technologies could evolve and become more prevalent in health care, it is crucial to ensure that these devices comply with existing legal frameworks and regulations [49,71]. HTA plays a pivotal role in evaluating the legal implications of AI-based medical devices by assessing factors such as data privacy, security, liability, and regulatory compliance [72].

One key legal aspect to consider is data privacy and protection [73,74]. AI-based medical devices often rely on vast amounts of patient data for training and decision-making. Therefore, it is essential to evaluate whether these devices adhere to relevant data protection laws, such as the General Data Protection Regulation in the European Union [75,76]. HTA examines the measures taken by AI-based medical device manufacturers to safeguard patient privacy, including data anonymization, encryption, and secure data storage practices [37]. These issues are highlighted in the Artificial Intelligence Act published in June 2023 by the European Commission [77,78].

Cybersecurity is another critical consideration [21,79]. As AI-based medical devices handle sensitive patient data and make critical health care decisions, it is crucial to assess the security measures implemented to prevent unauthorized access, data breaches, or tampering [80]. HTA evaluates the robustness of the security protocols implemented by device manufacturers and their compliance with industry standards and regulations [21,81].

Liability is also a significant legal aspect to be addressed in the context of AI-based medical devices [82,83]. When errors or adverse events occur due to the use of these devices, determining liability can be complex [84]. HTA examines the legal frameworks and liability guidelines pertaining to AI technologies, including whether clear guidelines exist regarding the responsibility of manufacturers, health care providers, and users in case of device malfunctions or errors [85]. Assessing liability aspects within the HTA process helps establish accountability and ensures that legal frameworks adequately address potential risks [86].

Regulatory compliance is a crucial consideration when assessing the suitability of AI-based medical devices for HTA [87]. Depending on the jurisdiction, AI devices need to undergo regulatory approval processes before being introduced into the market [88]. HTA examines whether AI-based medical device manufacturers have obtained the necessary regulatory approvals, such as clearance from the relevant health authorities or certification from regulatory bodies such as the Food and Drug Administration (FDA) in the United States or notified bodies in Europe [89]. This evaluation ensures that AI-based medical devices comply with existing regulations and are fit for clinical use [61].

## Discussion

### Recommendations to Adapt the 9 HTA Domains for AI-Based Medical Devices

Taking into account the previous considerations, some recommendations could be proposed to adapt and personalize these standard HTA domains to AI-based medical devices’ specificities. Therefore, we suggest 4 main recommendations by domain in Table 3.

**Table 3:** Recommendations to adapt and personalize the health technology assessment (HTA) of standard HTA domains to artificial intelligence (AI)–based medical device specificities.

Domains	4 recommendations per domain	Application examples
1 and 2—health problem, description, and technical characteristics of the technology	Assess the technical performance and reliability of the AI-based medical device, especially for ML^a^- and DL^b^-based medical devices Evaluate the usability and user interface of the AI-based medical device Assess the interoperability and compatibility of the AI-based medical device with existing systems Regularly update the assessment to reflect AI technological advancements Update the assessment periodically to incorporate new information and advancements related to the AI-based medical device	Consider the example of an AI-based DMS^c^ that assists patients with diabetes in managing their condition by providing blood glucose predictions based on their planned actions, thus contributing to better decision-making. The description and technical features involve defining patient data collection, data preprocessing, selecting relevant features, constructing an AI model, training the model & validating it using separate data, deploying it to provide blood glucose predictions to patients, and continuously improving it through real-time data collection.
3—safety	Evaluate AI compliance with safety regulations and standards and AI risk management processes Evaluate the AI’s robustness and resilience to errors or failures Assess the AI’s impact on patient safety, user safety, and long-term safety Consider the durability of the AI technology; regularly monitor and update safety information; and regularly monitor and reassess the AI’s safety profile incorporating new information, evidence, and user experiences	The potential risk of patient injury of an insulin delivery AI-based medical device system should be taken into account in the risk management process. In the case of an evolutive DL–based medical device without continuous ongoing safety assessment, a wrong dosage administration due to an AI error could provoke a serious adverse event (ie acid ketosis coma for a patient with diabetes). A risk management plan should be available and regularly updated with AI changes impact on safety plan.
4—clinical effectiveness	Compare the AI technology to existing alternatives or standard of care Assess the generalizability of the clinical evidence and analyze the clinical outcomes in subpopulations Consider patient-reported outcomes, quality-of-life measures, and impact on clinical workflow Regularly update the clinical evidence base of the AI-based medical devices and monitor consistency during the AI lifetime	The FDA^d^ in the United States has proposed a regulatory framework for modifications to evolutive AI- and ML-based software as a medical device with modification guidance focused on the risk to users and patients resulting from the AI changes. They have asked for an Algorithm Change Protocol with specific methods in place to achieve and control the risks of the anticipated types of modifications related to performance, use, or inputs. A continuous clinical effectiveness assessment could be an interesting approach for an AI-based medical device diabetes foot ulcer detection system for patients needing adaptative treatment modifications to prevent ulcer development.
5—costs and economic evaluation	Assess the budget impact of adopting the AI-based medical device within the health care system If needed, conduct a comprehensive cost-effectiveness analysis of the AI-based medical device compared to standard care and assess long-term impact on health care resource use (hospital admissions, length of stay, and time to diagnosis), health care disparities, and access to care in case of evolution of the AI’s performance Explore potential reimbursement strategies and work with payers to develop reimbursement models that align with the value and impact of the AI device (on clinical effectiveness, cost-effectiveness, and long-term sustainability) Establish mechanisms for ongoing monitoring and reassessment of the AI device’s economic value to ensure that it remains aligned with evolving health care priorities and resource allocation strategies	To evaluate a diabetic retinopathy screening AI-based medical device, clinical effectiveness is not sufficient. The cost-effectiveness of different types of AI diabetic retinopathy screening should be compared with no screening and ophthalmologist screening. A recent study demonstrated that AI-based screening was the most cost-effective, not only saving costs but also improving the quality of life of patients with diabetes. In this case, the long-term assessment of the economic impact of AI introduction in diabetic retinopathy screening highlighted the added value of this technology.
7—organizational aspects	Assess the organizational readiness and capacity for implementing AI-based medical devices in health care settings Conduct a thorough analysis of the impact of implementing AI-based medical devices on health care workflows and processes Establish clear guidelines and protocols for the appropriate use and integration of AI-based medical devices Monitor and evaluate the impact of AI-based medical device implementation on patient outcomes, quality of care, and patient satisfaction Regularly review and update implementation strategies based on the findings from monitoring AI	Decision makers and technology advocates need to address the complexities of AI more comprehensively and understand the systemic challenges that its adoption poses to health care organizations and systems. As an example, consider an AI tool used for diagnosing diabetic retinopathy in a primary care setting, such as by a family physician or nurse. In theory, this could lead to shorter waiting times for patients. However, if the health care organization faces challenges such as a shortage of specialized staff (eg, ophthalmologists), insufficient organization of care pathways, and lack of specialized facilities for proper management and follow-up after diagnosis, the introduction of AI might adversely affect the quality of care and patient experience.
Domains 6, 8, and 9—ethical analysis or patients and social aspects or legal aspects	Conduct an ethical analysis and evaluate the social implications of implementing the AI-based medical devices Assess the legal and regulatory aspects related to the AI-based medical device Promote transparency, interpretability, and explainability of the AI-based medical devices Monitor and address emerging ethical, social, and legal issues related to the AI-based medical devices Establish mechanisms for ongoing surveillance and evaluation of the AI device’s ethical, social, and legal implications	AI-based continuous glucose monitoring and insulin pumps should also be assessed on ethical and legal aspects. The risks of data theft and privacy breaches necessitate careful consideration of ethical and legal issues for patients. Although AI can aid in decision-making, it cannot wholly substitute a physician’s expertise. Effective regulations and systems designed to ensure safety, reduce bias, and enhance transparency are essential.

^a^ML: machine learning.

^b^DL: deep learning.

^c^DMS: diabetes management system.

^d^FDA: Food and Drug Administration.

**Figure 2:**
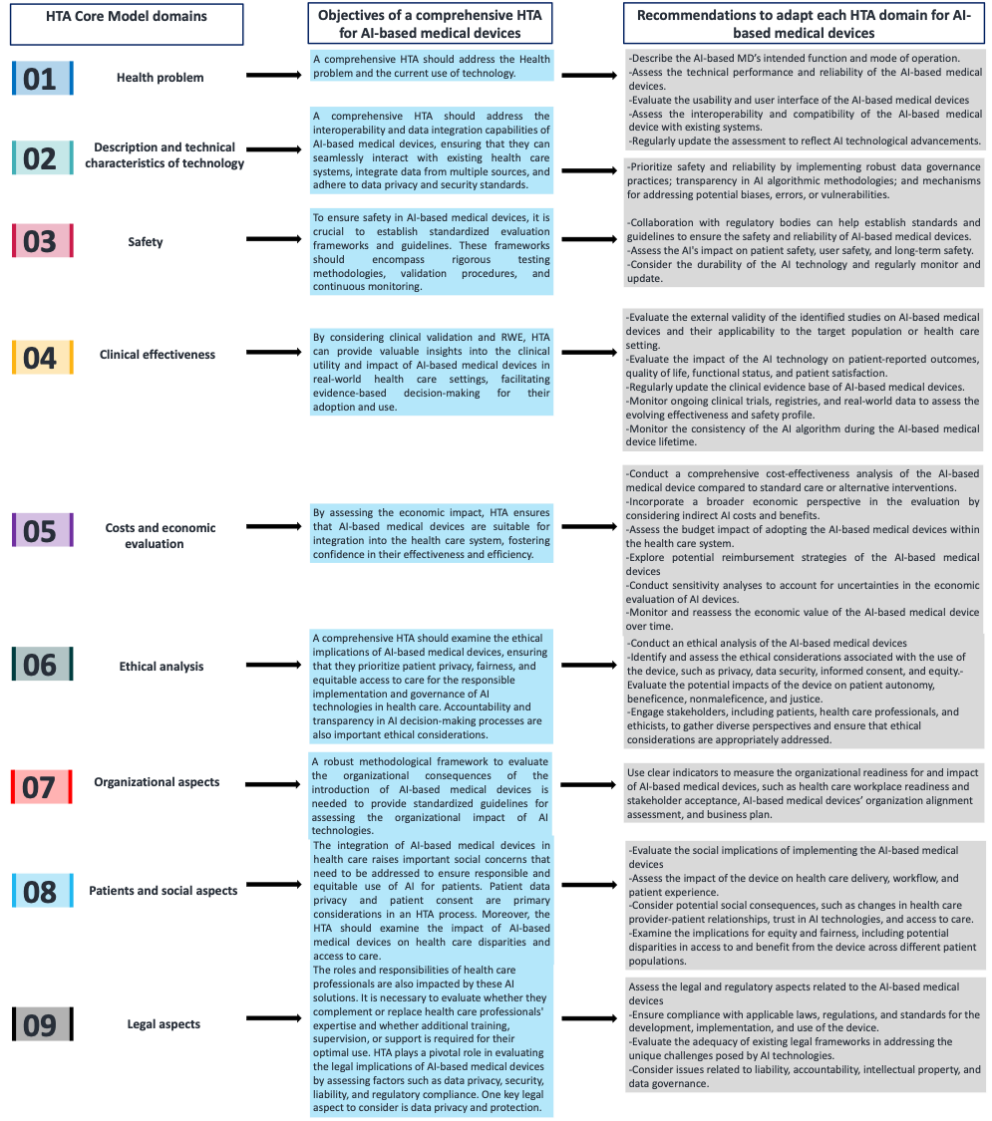
Suggested recommendations to adapt each standard health technology assessment (HTA) domain for the evaluation of artificial intelligence (AI)–based medical devices (MDs). RWE: real-world evidence.

The adaptation of the HTA process through a methodological framework for AI-based medical devices enhances the comparability of results across different evaluations and jurisdictions [90-92]. It promotes consistency in the assessment methodologies, reporting formats, and presentation of findings, enabling decision makers to make informed choices based on reliable and comparable evidence. This standardization contributes to the overall credibility and acceptance of HTA outcomes related to AI-based medical devices [93]. In addition, a methodological framework would address the need for appropriate expertise and skills to conduct the HTA of AI-based medical devices [94-99]. It would outline the qualifications and competencies required for the individuals involved in the assessment, including knowledge of AI technologies [46]. By defining the necessary expertise, the framework supports the development of a skilled workforce capable of conducting robust and reliable HTAs of AI-based medical devices (Figure 2).

Evaluating the trade-offs and weighing different features of AI-based medical devices is indeed a complex and important task, especially in domains such as health care where the impact on human lives is significant. The acceptability of AI-based medical devices should be assessed on a case-by-case basis considering various factors, including, for instance, performance, accuracy, cost, explainability, and the specific context in which they are being used.

Trade-offs between accuracy and cost are common in AI. It may be acceptable for AI to increase follow-up care costs if it significantly improves accuracy and patient outcomes. For example, if an AI system can detect diseases at an earlier stage, it might lead to more effective treatment and, ultimately, lower overall health care costs in the long run.

The balance between explainability and performance is a critical consideration. While explainable algorithms are preferred for safety and transparency reasons, there may be cases in which a highly complex, unexplainable algorithm outperforms explainable ones. In such cases, the trade-off between transparency and performance should be carefully evaluated based on the specific use case and potential risks.

The minimum performance for adding this technology to the tools available to human clinicians depends on the specific task and the level of trust that patients, health care providers, and regulators have in the technology. Some key factors to consider include the following: (1) The complexity of the task—AI-based medical devices should excel in tasks that are well defined and data driven, but they may not replace human clinicians in tasks requiring complex decision-making, empathy, or ethical considerations. (2) Safety and reliability—AI-based medical devices should demonstrate a high level of safety and reliability, ideally surpassing the performance of human clinicians in terms of avoiding errors. (3) Ethical considerations—AI-based medical devices should adhere to ethical standards, including patient privacy, informed consent, and unbiased decision-making, which are often considered even more important than performance metrics. (4) Regulatory approval—regulatory bodies often establish performance thresholds for AI-based medical devices. Compliance with these thresholds is essential for market acceptance.

In general, AI should aim to complement and enhance the capabilities of human clinicians rather than completely replacing them. The specific threshold for acceptable performance will vary across applications and contexts, and it should be determined through a combination of rigorous testing, peer-reviewed studies, and input from health care professionals and patients.

It is important to note that ethical considerations, patient safety, and the potential for bias should always be at the forefront of these discussions, and AI-based medical devices should not be adopted solely for the sake of automation or cost reduction if they compromise these critical aspects of health care.

### Use Case of the Application of the Aforementioned HTA Recommendations for AI: AI-Based Medical Devices in Pathways for Patients With Diabetes

Concerning the HTA domain 3 on the safety of an AI-based medical device, the potential risk of patient injury of an insulin delivery AI-based medical device system should be taken into account in the risk management process of algorithm development [96]. In the case of an evolutive deep learning–based medical device without continuous ongoing safety assessment, a wrong dosage administration due to an AI error, for instance, could provoke a serious adverse event such as an acid ketosis coma for a patient with diabetes.

A risk management plan should be available for users and regularly updated with the impact of the AI changes on the safety plan.

In relation to the HTA domain 4, which focuses on the effectiveness of an AI-based medical device, the FDA in the United States has proposed a regulatory framework for modifications to evolutive AI- and machine learning–based software as a medical device with modification guidance focused on the risk to users or patients resulting from the AI changes [100]. They have asked for an Algorithm Change Protocol with specific methods in place to achieve and control the risks of the anticipated types of modifications related to performance, use, or inputs. A continuous clinical effectiveness assessment could be an interesting approach for an AI-based medical device detection system for diabetes foot ulcer for patients needing adaptative treatment modifications to prevent ulcer development [101].

Concerning the HTA domain 5, to evaluate a diabetic retinopathy screening AI-based medical device, clinical effectiveness is not sufficient. The cost-effectiveness of different types of AI diabetic retinopathy screening should be compared with no screening and ophthalmologist screening. A recent study demonstrated that AI-based screening was the most cost-effective, not only saving costs but also improving the quality of life of patients with diabetes [44]. In this case, the long-term assessment of the economic impact of AI introduction in diabetic retinopathy screening highlighted the added value of this technology.

The HTA domain 7 on organizational impact has to assess how the AI-based medical device can be effectively integrated into the health care pathway and prevent wasteful spending. More thorough attention must be paid to the following aspects: (1) evaluating needs and determining the added value of the implementation of the AI-based medical device; (2) assessing workplace preparedness, including stakeholder acceptance of the introduction of the AI-based medical device and involvement; and (3) analyzing the alignment between AI technology and organizational structure [64]. Decision makers and technology advocates need to address the complexities of AI more comprehensively and understand the systemic challenges that its adoption poses to health care organizations and systems. As an example, consider an AI tool used for diagnosing diabetic retinopathy in a primary care setting, such as by a family physician or nurse [102]. In theory, this could lead to shorter waiting times for patients. However, if the health care organization faces challenges such as a shortage of specialized staff (eg, ophthalmologists), insufficient organization of care pathways, and lack of specialized facilities for proper management and follow-up after diagnosis, the introduction of AI might adversely affect the quality of care and patient experience. In such a scenario, the AI application might merely transfer the delay from primary to secondary care, failing to address the fundamental issue.

Finally, concerning the last domains (6, 8, and 9) about ethical, patient, social, and legal aspects, AI-based continuous glucose monitoring and insulin pumps should also be assessed on ethical and legal aspects [103]. While citizen juries have generally shown support for AI in research and treatment, concerns remain. The risks of data theft and privacy breaches necessitate careful consideration of ethical and legal issues for patients. Although AI can aid in decision-making, it cannot wholly substitute a physician’s expertise. Effective regulations and systems designed to ensure safety, reduce bias, and enhance transparency are essential.

By implementing these recommendations, stakeholders can foster the responsible integration, regulation, and evaluation of AI-based medical devices. These measures can enhance the evidence base, address ethical concerns, and maximize the potential benefits of these AI technologies in improving health care outcomes while protecting patient safety, privacy, and equity.

### Practical Challenges and Potential Barriers to Implementing HTAs Specific for AI-Based Medical Devices

When it comes to implementing specialized HTAs for AI-based medical devices, several practical challenges and potential barriers could be encountered and should be taken into account: First, AI technological evolution timeline—AI technologies evolve at a much faster pace compared to traditional medical devices, making it challenging for HTA frameworks to keep up with the latest developments and assess their long-term impact effectively. AI-based medical devices have a short product lifetime, between 12 and 18 months, in contrast to drug products. This shorter life cycle highlights the need for evolutive and fast-track HTA processes for AI-based medical devices. Second, data requirements and quality—AI systems rely heavily on large data sets for training and validation. Ensuring the availability of high-quality, representative data is a significant challenge. There is also the issue of data privacy and security, which must be addressed. The availability and quality of data and evidence required for conducting HTAs on AI-based medical devices present a complex and evolving landscape. Assessing the feasibility and challenges associated with gathering such data is crucial for robust evaluations. First, the availability of data can vary significantly depending on the AI-based medical device. While some devices may have access to vast amounts of high-quality real-world patient data, others might face limitations due to the novelty of the technology or issues related to data privacy. Second, the quality of data is paramount as inaccurate or biased data can lead to flawed assessments. Ensuring data accuracy, representativeness, and relevance is a constant challenge in AI-based medical device evaluations [104]. The rapid pace of AI development can result in limited long-term data, making it difficult to assess the device’s sustained performance and safety [105]. Balancing the need for robust evidence with the dynamic nature of AI technologies is a significant challenge that HTA organizations must address to provide valuable insights for informed decision-making in health care. Third, complexity and transparency—the complex algorithms used in AI-based medical devices can be difficult to understand and assess, leading to issues with transparency and explainability [17]. This complexity can pose a challenge for regulators and assessors in HTA processes. Fourth, clinical validation and safety requirements—generating robust clinical evidence to demonstrate the safety, efficacy, and effectiveness of AI-based medical devices can be challenging. This includes proving that these devices perform consistently across diverse patient populations. Moreover, to account for a life cycle that would include updates that may improve performance, one solution to investigate could be the proposition of the FDA in the United States in 2019 to experiment with a “dynamic certification” [100]. It allows for the re-evaluation of the AI-based medical device in case of a substantial modification of the indication of the medical device or the way to deliver the diagnosis, for instance. Fifth, regulatory and ethical considerations—adapting existing regulatory frameworks to accommodate AI-based medical devices, addressing ethical concerns such as bias, and ensuring equitable access are critical challenges [106]. Sixth, economic evaluation—determining the cost-effectiveness of AI-based medical devices, especially when benefits might be indirect or long term, poses a unique challenge for HTA [93]. Seventh, stakeholder engagement and trust—building trust among health care providers, patients, and policy makers regarding the reliability, trustworthiness, and usefulness of AI-based medical devices is crucial but challenging [107]. Eighth, integration into health care systems and interoperability—the integration of AI-based medical devices into existing health care workflows and systems can be complex and resource intensive [18]. Ninth, global and local applicability—ensuring that AI-based medical devices are effective and appropriate for use in different global and local contexts, considering varying health care systems and population needs, is another significant barrier.

Practically, one suggestion to implement such frameworks could be to implement these recommendations in the future European clinical joint assessment guidelines. As they are being currently discussed in a European project on a common HTA process for connected medical devices that includes AI-based medical devices, it could be the opportunity to tackle these challenges at the European level [108].

Addressing these challenges requires a concerted effort from regulators, health care providers, technology developers, and other stakeholders in the health care ecosystem.

### Conclusions

AI-based medical devices have the potential to transform health care delivery, but the suitability of the current comprehensive HTA requires careful adaptation of the evaluation across the 9 dimensions. While these AI devices show promise in improving accuracy, safety, and efficiency, there is a need for robust clinical validation, integration into workflows, economic evaluation, and addressing of ethical and legal implications. A comprehensive adapted HTA framework for AI-based medical devices can provide valuable insights into their effectiveness, cost-effectiveness, and societal impact, guiding their responsible implementation and maximizing their benefits for patients and health care systems.

## Appendix

Multimedia Appendix 1 The 9 health technology assessment (HTA) domains for the assessment of artificial intelligence–based medical devices adapted from the HTA Core Model proposed by the European Network for Health Technology Assessment.

Multimedia Appendix 2 Summary results of the scoping review and general characteristics of the studies included in the scoping review.

## Abbreviations

AI: artificial intelligence
FDA: Food and Drug Administration
HTA: health technology assessment
PRISMA: Preferred Reporting Items for Systematic Reviews and Meta-Analyses
RWE: real-world evidence

## Data Availability

The data sets generated during and analyzed during this study are available from the corresponding author on reasonable request.
