# Supplementary material for: Suitability of the Current Health Technology Assessment of Innovative Artificial Intelligence-Based Medical Devices: Scoping Literature Review
Source: J Med Internet Res. 2024 May 13;26:e51514. doi: 10.2196/51514 (PMC11130781; doi:10.2196/51514)
Supplement: Multimedia Appendix 1 [file jmir_v26i1e51514_app1.docx]

**Supplementary file 1 - Table 1**

**Nine health technology assessment domains for the assessment of AI-based medical devices, adapted from the Health Technology Assessment (HTA) Core Model® proposed by European network HTA***

| **HTA Domain** | **Title** |
| --- | --- |
| *Domain 1* | Health problem and current use of the technology |
| *Domain 2* | Description and technical characteristics of technology |
| *Domain 3* | Safety |
| *Domain 4* | Clinical effectiveness |
| *Domain 5* | Costs and economic evaluation |
| *Domain 6* | Ethical analysis |
| *Domain 7* | Organizational aspects |
| *Domain 8* | Patients and Social aspects |
| *Domain 9* | Legal aspects |

*Reference: HTA Core Model® - EUnetHTA. 2018.https://www.eunethta.eu/hta-core-model/ (accessed 3 Jun2023)
