# Supplementary material for: Suitability of the Current Health Technology Assessment of Innovative Artificial Intelligence-Based Medical Devices: Scoping Literature Review
Source: J Med Internet Res. 2024 May 13;26:e51514. doi: 10.2196/51514 (PMC11130781; doi:10.2196/51514)
Supplement: Multimedia Appendix 2 [file jmir_v26i1e51514_app2.docx]

**Supplementary file 2: Table 2:**

**Summary results of the scoping review and general characteristics of the studies included in the scoping review**

| **General characteristics of the studies included in the scoping review** | | | | |
| --- | --- | --- | --- | --- |
| Authors | **Publication date** | **Journal** | **Objective** | **HTA Assessment Domain for AI-based MDs related to each article** |
| Bajwa J. et al. | 2021 | Future Healthc J | To examine how artificial intelligence (AI) is transforming the practice of medicine and to discuss the implications of AI in healthcare. | Domains 3 & 4 |
| Bohr A. et al. | 2020 | Artif Intell Healthc | To explore the emergence and rise of AI in healthcare applications and its potential impact on the sector. | Domains 3 & 4 |
| Farah L. et al. | 2023 | Artif Intell Med | To evaluate whether current clinical studies on AI-based medical devices are comprehensive enough to support a full health technology assessment. | Domains 3 & 4, 5, 7 |
| Chen Y. et al. | 2022 | Integr Med Res | To discuss if the principles of health technology assessment and economic evaluation are applicable to traditional medicine. | Domain 5 |
| He J. et al. | 2019 | Nat Med | To provide insights into the practical implementation of AI technologies in medicine. | Domains 1 & 2, 7 |
| Farah L. et al. | 2023 | Mayo Clin Proc Digit Health | To assess the performance, interpretability, and explainability of AI-based health technologies and to inform healthcare stakeholders about what they need to know | Domains 1 & 2, 6&8 |
| Lehne M. et al. | 2019 | NPJ Digit Med | To argue why digital medicine requires interoperability to succeed. | Domain 1 & 2 |
| Esmaeilzadeh P. et al. | 2020 | BMC Med Inform Decis Mak | To investigate consumers' perspectives on the use of AI-based tools for healthcare purposes. | Domain 1 & 2 |
| Zia A. et al. | 2022 | J Pers Med | To delve into the application of AI in medical data mining. | Domain 1 & 2 |
| Ming J. et al. | 2022 | Cost Eff Resour Alloc | To provide an overview of the current landscape, challenges, and future directions in health technology assessment of medical devices. | Domains 1 & 2, 3& 4, 6&8, 9 |
| Filkins BL. et al. | 2016 | Am J Transl Res | To discuss privacy and security concerns in digital health and what translational researchers should know and do about it. | Domain 1 & 2 |
| Johnson KB. et al. | 2021 | Clin Transl Sci | To explore the implications of precision medicine and AI for personalized health care. | Domains 3&4,5 |
| Sloane EB. et al. | 2020 | Clin Eng Handb | To examine the role of AI in medical devices and clinical decision support systems. | Domains 3 & 4 |
| Steuten LMG. et al. | 2016 | OMICS J Integr Biol | To assess the early stages of health technology assessment for precision biomarkers in oral health and systems medicine. | Domains 3 & 4 |
| Javaid M. et al. | 2022 | Int J Intell Netw | To highlight the significance of machine learning in healthcare and discuss its features, pillars, and applications. | Domains 3 & 4 |
| Rowland SP. et al. | 2020 | NPJ Digit Med | To evaluate the clinical value of mobile health (mHealth) for patients. | Domains 3 & 4 |
| Voets MM. et al. | 2022 | Value Health J Int Soc Pharmacoeconomics Outcomes Res | To conduct a systematic review of health economic evaluations focused on AI in healthcare. | Domains 3 & 4 |
| Kirisits A. et al. | 2013 | Appl Health Econ Health Policy | To address the economic evaluation challenges ahead for medical devices. | Domains 3 & 4 |
| Park SH, et al. | 2021 | Korean J Radiol | To discuss the key principles of clinical validation, device approval, and insurance coverage decisions for artificial intelligence in healthcare. | Domains 3 & 4 |
| Tsopra R, et al. | 2021 | BMC Med Inform Decis Mak | To propose a framework for validating AI technologies in precision medicine, considering insights from the European ITFoC consortium. | Domains 3 & 4 |
| Bolboacă SD | 2019 | Comput Math Methods Med | To review the anatomy of medical diagnostic tests, the phases involved, and the statistical treatment of data. | Domains 3 & 4 |
| Hogervorst MA, et al. | 2022 | Front Pharmacol | To examine the use of real-world data in health technology assessment, particularly for complex health technologies. | Domains 3 & 4 |
| Simon GE, et al. | 2022 | Clin Pharmacol Ther | To discuss when real-world data can be trusted for evaluating new medical treatments. | Domains 3 & 4 |
| Pongiglione B, et al. | 2021 | Int J Technol Assess Health Care | To evaluate if existing real-world data sources are suitable for HTA of medical devices in Europe. | Domains 3 & 4 |
| Pongiglione B, Torbica A | 2022 | Health Econ | To explore the potential of routinely collected administrative data for generating real-world evidence for medical device evaluation. | Domains 3 & 4 |
| Zemplényi A, et al. | 2023 | Front Public Health | To offer recommendations for overcoming barriers in using AI-driven evidence in health technology assessment. | Domains 3 & 4, 5, 9 |
| Daubner-Bendes R, et al. | 2020 | Front Public Health | To discuss the methodological challenges and recommendations for HTA of medical devices in Central and Eastern Europe. | Domains 3 & 4 |
| Larson DB, et al. | 2021 | J Am Coll Radiol | To summarize and recommend regulatory frameworks for the development and evaluation of AI-based diagnostic imaging algorithms. | Domains 3 & 4 |
| Choudhury A, Asan O | 2020 | JMIR Med Inform | To review the role of AI in improving patient safety outcomes. | Domains 3 & 4 |
| Belenguer L | 2022 | AI Ethics | To explore discriminatory algorithmic decision-making models in AI and propose machine-centric solutions adapted from the pharmaceutical industry. | Domains 3 & 4 |
| Binder L, et al. | 2022 | Curr Oncol | To assess the impact of changes in the Health Technology Assessment process for oncology drugs on public payer reimbursement recommendations. | Domain 5 |
| Wolff J, et al. | 2020 | J Med Internet Res | To systematically review the economic impact of AI in healthcare. | Domain 5 |
| Abràmoff MD, et al. | 2022 | NPJ Digit Med | To propose a reimbursement framework for healthcare AI technologies. | Domain 5 |
| Bélisle-Pipon J-C, et al. | 2021 | Front Artif Intell | To discuss what makes AI exceptional in health technology assessment. | Domains 5, 6 & 8 |
| Alami H, et al. | 2020 | J Med Internet Res | To anticipate the complexities that artificial intelligence brings to health technology assessment. | Domain 5, 6&8 |
| Love-Koh J, et al. | 2018 | PharmacoEconomics | To evaluate the potential impacts of precision medicine on health technology assessment. | Domain 5 |
| Gomez Rossi J, et al. | 2022 | JAMA Netw Open | To assess the cost-effectiveness of AI as a decision-support system in the detection and grading of melanoma, dental caries, and diabetic retinopathy. | Domain 5 |
| Naik N, et al. | 2022 | Front Surg | To discuss legal and ethical considerations of AI in healthcare and who is responsible. | Domains 6 & 8, 9 |
| Haynes CL, et al. | 2007 | J Med Ethics | To explore the legal and ethical considerations of processing patien -identifiable data without consent. | Domains 6 & 8 |
| McCradden MD, et al. | 2020 | Lancet Digit Health | To address the ethical limitations of algorithmic fairness solutions in healthcare machine learning. | Domains 6 & 8 |
| Gianfrancesco MA, et al. | 2018 | JAMA Intern Med | To identify potential biases in machine learning algorithms using electronic health record data. | Domains 6 & 8 |
| Fletcher RR, et al. | 2021 | Front Artif Intell | To address fairness, bias, and the appropriate use of AI and machine learning in global health. | Domains 6 & 8 |
| Tachkov K, et al. | 2022 | Front Public Health | To identify barriers to using AI methodologies in health technology assessment in Central and East European countries. | Domains 6 & 8 |
| Durán & Jongsma et al. | 2021 | J Med Ethics | The objective is to explore the epistemological and ethical foundations of trust in medical AI, particularly addressing the fear of opaque algorithmic processes. | Domains 6 & 8 |
| Amann et al. | 2020 | BMC Med Inform Decis Mak | To provide a multidisciplinary perspective on explainability in AI for healthcare, suggesting that comprehensibility of AI systems is crucial for their ethical and practical integration into clinical practice. | Domains 6 & 8 |
| Kiseleva et al. | 2022 | Front Artif Intell | To provide input on the transparency of AI in healthcare, describing it as a multilayered system of accountabilities, and discusses the balance between legal requirements and technical limitations. | Domains 6 & 8 |
| Baltaxe et al. | 2023 | J Med Internet Res | To assess medical device software used in chronic patient care at a tertiary hospital, aiming to evaluate its support of health care services. | Domains 6 & 8 |
| Garfield et al. | 2016 | Value Health J Int Soc Pharmacoeconomics Outcomes Res | to addressthe practices, challenges, and recommendations for health technology assessment (HTA) of molecular diagnostics within the context of medical devices and diagnostics. | Domains 6 & 8 |
| Fraser et al. | 2023 | Expert Rev Med Devices | The paper reviews definitions, expert recommendations, and regulatory initiatives regarding AI in medical device software and high-risk medical devices. | Domains 6 & 8 |
| Beckers et al. | 2021 | Phys Medica PM | To discuss the implications of the EU medical device regulation for AI-based medical device software in medical physics. | Domains 6 & 8, 9 |
| Melvin & Torre et al. | 2019 | EFORT Open Rev | To provide insights from the regulator’s perspective on new medical device regulations. | Domains 6 & 8 |
| Fleetcroft et al. | 2021 | BMJ Surg Interv Health Technol | To suggest use of the IDEAL framework as a guide to design clinical device studies in accordance with the new European Medical Device Regulation. | Domains 6 & 8 |
| Alami et al. | 2020 | J Health Organ Manag | To investigate organizational readiness for AI in healthcare, providing insights for decision-making and practice. | Domain 7 |
| Segur-Ferrer et al. | 2022 | JMIR Res Protoc | To outline methodological frameworks and dimensions for digital health technology assessment. | Domain 7 |
| Weinert et al. | 2022 | JMIR Med Inform | To analyze the perspective of IT decision-makers on factors influencing the adoption and implementation of AI technologies in German hospitals. | Domain 7 |
| de Hond et al. | 2022 | NPJ Digit Med | To provide a scoping review of guidelines and quality criteria for AI-based prediction models in healthcare. | Domain 7 |
| Ahuja et al. | 2019 | PeerJ | To examine the impact of AI in medicine on the future role of physicians. | Domain 9 |
| Widrig & Tag et al. | 2014 | Int J Technol Assess Health Care | To propose a framework for identifying legal issues in health technology assessment. | Domain 9 |
| Vella Bonanno et al. | 2019 | Expert Rev Pharmacoecon Outcomes Res | To reflect on opinions of policymakers, payers, and academics in the field of HTA concerning a proposal for a regulation on HTA in Europe. | Domain 9 |
| McKee & Wouters et al. | 2022 | Int J Health Policy Manag | To discuss the challenges of regulating AI in healthcare. | Domain 9 |
| Hordern et al. | 2016 | Eur J Health Law | To delve into data protection compliance in the context of digital health. | Domain 9 |
| Stanberry et al. | 1998 | J Telemed Telecare | To discuss data protection, security, and European law in relation to telemedicine. | Domain 9 |
| Poullet et al. | 1991 | Stud Health Technol Inform | To address legal aspects of data protection in medical informatics. | Domain 9 |
| Dove & Chen et al. | 2020 | J Law Med Ethics J Am Soc Law Med Ethics | To discuss the extent to which the EU General Data Protection Regulation applies to citizen scientist-led health research with mobile devices. | Domain 9 |
| Marovic & Curcin et al. | 2020 | JMIR Med Inform | To examine the impact of GDPR on health data management in Serbia, a European Union candidate country. | Domain 9 |
| European commission | 2023 | European commission website | To promote regulation on AI at a european level | Domain 9 |
| European parliament | 2021 | Eureopean parliament website | To act harmonised rules on AI in European Union | Domain 9 |
| Camara et al. | 2015 | J Biomed Inform | To lead a survey focusing on security and privacy issues in implantable medical devices. | Domain 9 |
| Migliore et al. | 2009 | Expert Rev Med Devices | To discuss the management of the introduction and use of medical devices in clinical practice in Italy, within the context of Health Technology Assessment. | Domain 9 |
| Pisapia et al. | 2022 | Clin Chem Lab Med | To discuss the novelties of the regulation on health technology assessment and its importance for EU health policies. | Domain 9 |
| MALIHA G et al. | 2021 | Milbank Quarterly | To discuss the balance between ensuring safety and fostering innovation in the application of artificial intelligence (AI) in medicine, with a focus on legal aspects and liability. | Domain 9 |
| Jassar S et al. | 2022 | Healthcare Management Forum | To seem to be to explore the future of AI in medicine from a legal perspective, particularly regarding the implications for health leaders. | Domain 9 |
| Samore MH et al. | 2004 | Journal of the American Medical Association | To discuss the surveillance of medical device-related hazards and adverse events in hospitalized patients, which may involve AI technology. | Domain 9 |
| Bleher H, et al. | 2022 | AI Ethics Journal | To investigate how responsibility is assigned when AI-driven clinical decision support systems are used, addressing the concept of diffused responsibility. | Domain 9 |
| Street J et al. | 2020 | International Journal of Technology Assessment in Health Care | To define the role of the public in Health Technology Assessment (HTA) and decision-making processes informed by HTA, potentially in the context of AI technologies. | Domain 9 |
| Massella M et al. | 2022 | Health Technology | To discuss regulatory considerations for the use of machine learning tools in clinical trials, indicating a focus on compliance and regulatory frameworks. | Domain 9 |
| Marcus HJ et al. | 2016 | The British Medical Journal | To seem to be an examination of the processes for regulatory approval of new medical devices, which may include AI-powered devices. | Domain 9 |
| Milam ME, et al. | 2023 | Clinical Radiology | To summarize the current status and future perspectives of FDA-approved AI tools in chest radiology within the United States. | Domain 9 |
